# Table Tennis for Health: A Multidimensional Perspective on Its Physical, Emotional, and Social Advantages

**DOI:** 10.3390/healthcare13182352

**Published:** 2025-09-18

**Authors:** Pilar Aparicio-Chueca, Noa Muñoz-Vila

**Affiliations:** Business Department, Faculty of Economics and Business, University of Barcelona, 08034 Barcelona, Spain

**Keywords:** table tennis, health promotion, physical activity, emotional health, emotional well-being

## Abstract

**Background/Objectives**: Table tennis is commonly perceived as a recreational or competitive sport; however, growing evidence highlights its potential as a multidimensional tool for health promotion. This study investigates the perceived physical, cognitive, emotional, and social benefits of regular table tennis practice, emphasizing its contribution to health beyond the purely sporting dimension. **Methods**: A mixed-method design with a predominantly quantitative approach was employed. A structured questionnaire was administered to 329 table tennis players in Catalonia. Descriptive statistics, exploratory factor analysis (EFA), multiple linear regression, Pearson correlations, and hierarchical cluster analysis (Ward’s method) were conducted to examine perceived benefits and identify user profiles. Factor analysis revealed two dimensions: physical–cognitive and emotional–social benefits. **Results**: The EFA produced a robust two-factor structure, explaining 76.6% of the variance (KMO = 0.941; Bartlett’s test, *p* < 0.001). Both dimensions showed excellent internal consistency (Cronbach’s α > 0.91). Regression analysis demonstrated that both factors significantly predicted the overall perception of table tennis as a health-enhancing activity (R^2^ = 0.199), with physical–cognitive benefits exerting the strongest effect (β = 0.375; *p* < 0.001). Cluster analysis identified three distinct profiles: Skeptical, Functional, and Integrative—with significant differences in perceived benefits (η^2^ = 0.710 for the emotional–social factor). **Conclusions**: Table tennis emerges as an inclusive, low-impact activity with strong potential to foster physical, emotional, and social well-being. Its accessibility and adaptability make it appropriate for diverse populations. These findings support its inclusion in public health strategies and community programs promoting holistic wellness. Future research should further explore motivational drivers across profiles and extend analyses to underrepresented populations.

## 1. Introduction

In recent decades, physical activity has assumed a central role in public health, serving both preventive and therapeutic functions in relation to a wide range of physical, mental, and social conditions. Within this framework, sport has become a key instrument for promoting well-being—not only because of its physical dimension, but also due to its positive effects on mental health, social interaction, and overall quality of life. However, most studies and initiatives have traditionally focused on mainstream sports, often overlooking less prominent disciplines that may nonetheless offer substantial health benefits. One such discipline is table tennis.

Table tennis, practiced worldwide at both recreational and competitive levels, is characterized by its accessibility, low cost, and adaptability across different ages and physical conditions. Although frequently associated with leisure or social play, regular practice has been shown to provide benefits in physical, cognitive, emotional, and social domains [1,2,3,4,5,6]. Despite these advantages, empirical research examining the comprehensive health impact of table tennis remains scarce.

Despite its limited media exposure, table tennis is a federated sport with a significant number of licensed players. With more than 350 million practitioners globally, it is one of the most widely played sports in the world. Its affordability, adaptability, and inclusive nature make it particularly suitable for individuals of all ages and physical or cognitive abilities.

In Spain, 19,347 players were registered in 2024 [7], reflecting an active community that combines both high-performance and amateur practice. This dual character positions table tennis as a versatile tool for health promotion in diverse contexts, ranging from competitive sport to social intervention [8,9].

Recent initiatives have incorporated table tennis into health-promotion programs, particularly to improve the quality of life of individuals with neurodegenerative conditions such as Parkinson’s and Alzheimer’s disease [10,11,12]. In Catalonia, for example, the Ganxets Club in Reus [13,14] has developed a program for people with Parkinson’s in collaboration with hospitals and universities, while CER L’Escala has organized therapeutic sessions for individuals with Parkinson’s and Alzheimer’s with the support of the public health system [15]. The Catalan Table Tennis Federation has also launched the Pingpongpertothom (PPxTT) initiative [16], which promotes inclusive events focusing on physical activity, emotional well-being, and social cohesion.

Beyond its competitive dimension, table tennis has been integrated into broader health initiatives. At Bellvitge University Hospital, for instance, it forms part of the BCN Salut Games, aimed at encouraging healthy habits among employees, patients, and the general population. At the national level, the AST Club, in collaboration with patient associations, has organized the *Table Tennis Training Campus* for individuals with Parkinson’s disease [17], representing a pioneering model of adapted performance training. Internationally, initiatives such as PingPongParkinson (active in over 25 countries) and the French program Ping4Alzheimer [18,19] have demonstrated benefits in memory, coordination, and mood, reinforcing the relevance of table tennis as a socio-health intervention strategy.

### 1.1. Physical Benefits and Body Condition

A growing body of research has highlighted the potential of table tennis to enhance physical fitness across the lifespan. Pluta et al. (2020) reported that the sport improves motor coordination, balance, and muscle strength—findings corroborated by longitudinal studies in both children [1,20,21,22,23,24] and older adults [2]. Additional benefits include increased bone density, lean muscle mass, and agility [25].

Compared with other racket sports such as tennis or badminton, table tennis exerts less stress on the joints, making it a safer option for children, individuals with joint-related conditions, and older adults [24]. Moreover, it requires less infrastructure than other aerobic activities such as swimming or cycling, while still offering substantial cardiovascular benefits [1,20,21,22,23,25].

### 1.2. Cognitive and Neurological Benefits

Table tennis demands the simultaneous engagement of attention, motor planning, and working memory, thereby providing significant cognitive stimulation. Neuroimaging studies have demonstrated enhanced brain connectivity in regular players, particularly in areas related to executive control, visual perception, and emotional regulation [25,26].

These effects are not limited to elite athletes; amateur players also exhibit cognitive improvements and reduced age-related decline [21,25]. Furthermore, interventions targeting children and adolescents with ADHD, ASD, or intellectual disabilities have reported gains in executive function, motor coordination, and visual perception following 8- to 16-week programs [27,28].

### 1.3. Emotional and Social Benefits

Beyond its physical and cognitive effects, table tennis provides significant emotional benefits [29,30]. It has been shown to enhance mood, reduce stress, and improve self-esteem. According to the Catalan Table Tennis Federation [16], it also serves as a preventive tool against conditions such as anxiety and depression. Clinical programs, such as those at the University Clinic of Manresa, have underscored their value in helping individuals disconnect from daily concerns and sustain concentration.

At the social level, table tennis fosters a sense of belonging and inclusion. Initiatives targeting older adults and vulnerable groups have demonstrated improvements in quality of life, community participation, and reductions in social isolation [27,31,32]. In this sense, table tennis should be understood not merely as a sport but as a valuable public health resource and a tool for social cohesion.

Building on these findings, the present study analyzes the perceptions of table tennis players regarding the physical, cognitive, emotional, and social benefits of the sport, with a specific focus on the Catalan context. The aim is to provide empirical evidence that positions table tennis as a comprehensive health-promoting activity, extending beyond its recreational or competitive dimensions.

## 2. Materials and Methods

### 2.1. Study Design

A structured online questionnaire was specifically designed for this study. A total of 329 individuals of different genders and age groups who regularly practiced table tennis completed the instrument.

The primary objective was to assess players’ perceptions of the physical, cognitive, emotional, and social benefits of table tennis, as well as to explore related aspects such as motivations, sporting background, and training frequency.

### 2.2. Statistical Analysis

All analyses were conducted using SPSS version 29 (IBM Corp., Armonk, NY, USA). Descriptive statistics, cross-tabulations, and mean comparisons were applied to examine differences and relationships among variables. To evaluate perceptions of benefits, several analytical techniques were employed:

- Exploratory Factor Analysis (EFA): Conducted using principal component extraction and Varimax rotation to reduce the 16 Likert-scale items into latent factors representing perceived benefits. Factor scores were subsequently computed.
- Regression Analysis: Factor scores were entered as predictors in a multiple linear regression model to explain the overall perception of table tennis as a health-promoting activity.
- Correlations: Pearson correlations were calculated between key variables.
- Cluster Analysis: A hierarchical cluster analysis (Ward’s method) was performed to identify perception profiles among participants.

This multi-method approach reduced the complexity of the measurement instrument and facilitated the identification of distinct analytical profiles.

### 2.3. Participants

The sampling strategy followed a voluntary or self-selection procedure, a form of non-probability sampling in which individuals decide to participate on their own initiative. This approach may introduce response bias, as participants with stronger opinions or greater interest are more likely to take part.

The study sample consisted of 329 table tennis practitioners (Table 1). The majority were over 50 years of age (50.3%), followed by those aged 15–20 (15%). In terms of playing experience, 22.8% reported more than 20 years of practice, 21.1% between one and three years, and 19.9% between 10 and 20 years. At the other extreme, 5.8% had less than one year of experience.

Regarding gender, 80.3% identified as male, 19.4% as female, and 0.3% as non-binary. Concerning place of practice, 89.3% trained at a club, while smaller proportions reported practicing at home, school, or high-performance centers.

Training frequency varied: 58.1% trained one to two days per week, 28.0% trained three to four days, 6.1% trained five or more days, and 7.8% reported not currently training. Most sessions (78.6%) lasted between one and two hours. Finally, 77.2% of respondents reported participation in competitions, reflecting a high level of engagement within the sample.

## 3. Results

An exploratory factor analysis (EFA) was conducted to examine the underlying structure of perceptions related to the practice of table tennis. The analysis included 16 items assessing the perceived benefits reported by practitioners. Preliminary indicators indicated excellent sampling adequacy: the KMO index reached 0.941, indicating high sampling adequacy, and Bartlett’s test of sphericity was significant (χ^2^ = 2196.804; df = 120; *p* < 0.001), confirming sufficient correlations among the items.

Using principal component extraction with Varimax rotation, two main factors with eigenvalues greater than 1 were identified, explaining 76.63% of the total variance.

- Factor 1 (39.52% of the variance) grouped items related to the *emotional and social* benefits of table tennis, such as mood improvement, self-esteem enhancement, promotion of social relationships, and feelings of inclusion and well-being. Factor loadings were high (several exceeding 0.80), confirming strong internal consistency.
- Factor 2 (37.11% of the variance) grouped items associated with *physical and cognitive* benefits, including improvements in coordination, reflexes, concentration, agility, and overall health. Factor loadings were similarly high, indicating a coherent and robust structure.

Internal consistency was assessed using Cronbach’s alpha. The physical–cognitive dimension (7 items) obtained an alpha of 0.919, while the emotional–social dimension (9 items) yielded an alpha of 0.947, indicating excellent reliability for both constructs. These findings confirm a clear bifactorial structure in the perception of table tennis benefits, validating the measurement instrument and providing insight into the mechanisms through which this activity contributes to health.

A multiple linear regression analysis was then performed using the overall perception of the relationship between table tennis and health (scale 1–10) as the dependent variable, and the factor scores as predictors. The model was significant (F = 15.074; df = 2.121; *p* < 0.001), explaining 19.9% of the variance (R^2^ = 0.199) (Table 2).

Both predictors were statistically significant: the physical–cognitive factor (β = 0.375, *p* < 0.001) and the emotional–social factor (β = 0.242, *p* = 0.003). Pearson correlations supported these findings, showing significant associations between overall perception and both factors: r = 0.375 (*p* < 0.001) for the physical–cognitive factor and r = 0.242 (*p* = 0.007) for the emotional–social factor (Table 3). No correlation was observed between the two factors (r = 0.000).

The regression results provide empirical evidence of the explanatory role of both dimensions in shaping the overall perception of table tennis as a health-promoting activity. Although the variance explained was modest (19.9%), both factors made meaningful contributions, with the physical–cognitive dimension emerging as the strongest predictor.

Finally, a hierarchical cluster analysis (Ward’s method) identified three distinct perception profiles. Cluster 3 reported the highest overall perception (M = 9.08), followed by Cluster 2 (M = 7.84) and Cluster 1 (M = 7.78). Significant differences were observed for both the emotional–social (F = 148.314; *p* < 0.001) and physical–cognitive (F = 81.405; *p* < 0.001) factors (Table 4).

This segmentation identified three meaningful groups: Skeptics, Functional, and Integrative. The Skeptics, who reported low scores on both dimensions, could benefit from targeted awareness campaigns. In contrast, the Integrative group demonstrated a comprehensive recognition of the potential of table tennis and may serve as valuable ambassadors in public health initiatives. The Eta^2^ values reinforced this interpretation, showing that the emotional–social factor was the most discriminating variable among profiles (η^2^ = 0.710).

## 4. Discussion

The findings of this study confirm the multidimensional value of table tennis as a health-promoting activity, with two primary domains “physical and cognitive“ and “emotional and social”—emerging from participants’ perceptions. These results are consistent with prior research highlighting the physical and neurological benefits of table tennis. For example, Naderi et al. [2,4] demonstrated improvements in bone health, muscle mass, and physical performance among older adults, while Wei et al. [10] and Yamasaki [6] emphasized its role in enhancing cortical connectivity and preventing cognitive decline. The present study extends these findings by showing that participants strongly associate table tennis with physical coordination, reflexes, and concentration.

Equally important, the findings underscore the influence of the emotional–social dimension, which exerted a strong effect on participants’ perceptions of table tennis as a health-enhancing activity. This aligns with the evidence of Olsson et al. [12] and Inoue et al. [11], who reported improvements in mood, motivation, and social interaction among individuals with Parkinson’s disease. Likewise, studies such as Hertting et al. [5] and To-Aj et al. [33] suggest that both recreational and competitive practice can foster well-being, social inclusion, and quality of life among working populations and older adults. Collectively, these findings indicate that the health impact of table tennis extends beyond physical benefits, highlighting its potential as a psychosocial intervention tool.

The cluster analysis contributes to the literature by identifying three distinct perception profiles. This segmentation aligns with earlier findings by Pradas et al. [1,21], who reported fitness outcomes varying by sex and age, and by González-Devesa et al. [29], who highlighted differential impacts in children and adolescents. Our results suggest that health-promotion programs may be more effective if adapted to the motivational profiles of participants—for example, by implementing awareness strategies for more skeptical groups or leveraging integrative profiles as ambassadors in community health campaigns.

Despite these promising results, several limitations must be acknowledged. First, reliance on self-reported data may have introduced subjective bias, as no objective health indicators (e.g., physical fitness tests, biomarkers, or clinical assessments) were included. Second, the sample was primarily recruited from table tennis clubs, potentially overrepresenting competitive or highly engaged players and underrepresenting casual practitioners or marginalized groups. Third, the age distribution was skewed toward older adults, limiting generalizability to children and adolescents. Finally, although the regression model was statistically significant, it accounted for only 20% of the variance, indicating moderate explanatory power.

Future research should therefore adopt mixed-method designs, integrate physiological and psychological measures, and broaden sampling across diverse demographics, particularly underrepresented populations such as individuals with disabilities or those outside formal club settings.

From a practical perspective, our findings suggest that table tennis represents a low-cost, safe, and adaptable activity that can be integrated into public health, educational, and workplace programs. Initiatives such as PingPongParkinson or Ping4Alzheimer illustrate their therapeutic potential for neurological conditions, while workplace-based interventions [5] highlight their capacity to promote well-being among employees. In this respect, table tennis may be regarded not only as a sport but also as a complementary practice that fosters social cohesion, stress reduction, and healthy aging.

In summary, this study contributes to the growing body of evidence positioning table tennis as an inclusive, multidimensional health-promoting activity. By integrating our findings with previous research, addressing limitations, and outlining practical applications, we provide a foundation for further investigation into its role within holistic health strategies.

## 5. Conclusions

This study demonstrates that table tennis holds significant potential as a health-promoting activity, providing benefits that extend from physical fitness to emotional and social well-being [1,2,3,4,5,6]. Its accessibility, low joint impact, and playful nature make it particularly suitable for individuals of diverse ages, skill levels, and physical conditions.

This research underscores the importance of recognizing table tennis not only as a competitive sport but also as a multidimensional activity with applications in both social and health contexts. Expanding and strengthening programs that incorporate table tennis may contribute to enhancing individual well-being and encouraging healthy lifestyles across different populations.

## Figures and Tables

**Table 1:** Description of the Study Sample.

	Frequency	Percentage
Age		
<15 years	20	5.8
15–20 years	52	15.0
21–25 years	26	7.5
26–30 years	14	4.0
31–40 years	22	6.4
41–50 years	38	11.0
>50 years	174	50.3
Gender		
Male	278	80.3
Female	67	19.4
Non-binary	1	0.3
How long have they been playing?		
<1 year	20	5.8
1–3 years	73	21.1
3–5 years	47	13.6
5–10 years	58	16.8
10–20 years	69	19.9
>20 years	79	22.8
Main place of practice		
Club	309	89.3
High-performance centre	9	2.6
School/Institute	1	0.3
Home	4	1.2
Others	23	6.6
Training frequency		
I don’t train	27	7.8
1–2 days	201	58.1
3–4 days	97	28.0
5 days or more	21	6.1
Training duration		
<1 h	22	6.4
1–2 h	272	78.6
2–3 h	50	14.5
>3 h	2	0.6
Do they currently compete?		
Yes	267	77.2
No	79	22.8

**Table 2:** Regression Model Summary.

Regression Model Summary									
Model	R	R Square	Adjusted R Square	Std. Error of the Estimate	Change Statistics				
					R^2^ Change	F Change	gl1	gl2	Sig. F
1	0.447	0.199	0.186	1.092	0.199	15.074	2	121	<0.001

**Table 3:** Regression Coefficients.

Model	Unstandardized Coefficients		Standardized Coefficients	t	Sig.
	B	Desv. Error	Beta		
(Constant)	8.581	0.098		87.511	<0.001
Emotional and Social Benefits	0.293	0.098	0.242	2.981	0.003
Physical and Cognitive Benefits	0.454	0.098	0.375	4.611	<0.001

**Table 4:** Cluster Means.

Clusters	Individual Perception of Table Tennis and Health	Emotional and Social Benefits	Physical and Cognitive Benefits
1	7.78	−1.782	−1.026
2	7.84	0.593	−1.046
3	9.08	0.182	0.678

## Data Availability

The data supporting the findings of this study are available from the corresponding author upon reasonable request. The dataset is not publicly available due to privacy restrictions.
